# What is beyond a qRT-PCR study on mesenchymal stem cell differentiation properties: how to choose the most reliable housekeeping genes

**DOI:** 10.1111/j.1582-4934.2012.01660.x

**Published:** 2013-01-11

**Authors:** Enrico Ragni, Mariele Viganò, Paolo Rebulla, Rosaria Giordano, Lorenza Lazzari

**Affiliations:** a Cell Factory “Franco Calori”, Center for Transfusion Medicine, Cellular Therapy and Cryobiology, Department of Regenerative Medicine, Fondazione IRCCS Ca' Granda Ospedale Maggiore PoliclinicoMilano, Italy

**Keywords:** regenerative medicine, mesenchymal stem cells, differentiation potency, qRT-PCR, housekeeping genes

## Abstract

In the last years, mesenchymal stem cells (MSCs) have been identified as an attractive cell population in regenerative medicine. In view of future therapeutic applications, the study of specific differentiation-related gene expression is a pivotal prerequisite to define the most appropriate MSC source for clinical translation. In this context, it is crucial to use stable housekeeping genes (HGs) for normalization of qRT-PCR to obtain validated and comparable results. By our knowledge, an exhaustive validation study of HGs comparing MSCs from different sources under various differentiation conditions is still missing. In this pivotal study, we compared the expression levels of 12 genes (*ACTB*, *β2M*, *EF1alpha*, *GAPDH*, *GUSB*, *PPIA*, *RPL13A*, *RPLP0*, *TBP*, *UBC*, *YWHAZ* and 18S rRNA) to assess their suitability as HGs in MSCs during adipogenic, osteogenic and chondrogenic differentiation. We demonstrated that many of the most popular HGs including 18S rRNA, *B2M* and *ACTB* were inadequate for normalization, whereas *TBP/YWHAZ*/*GUSB* were frequently identified among the best performers. Moreover, we showed the dramatic effects of suboptimal HGs choice on the quantification of cell differentiation markers, thus interfering with a reliable comparison of the lineage potential properties among various MSCs. Thus, in the emerging field of regenerative medicine, the identification of the most appropriate MSC source and cell line is so crucial for the treatment of patients that being inaccurate in the first step of the stem cell characterization can bring important consequences for the patients and for the promising potential of stem cell therapy.

## Introduction

In the recent years, human adult mesenchymal stem cells (MSCs) and tissue engineering technology have gained a crucial role for the treatment of human diseases. The most intriguing goal of regenerative medicine is to rapidly translate cutting-edge stem cell laboratory research into the clinical and commercial arenas to replace or repair diseased tissues and organs [1–3]. The choice of the optimal source of MSCs for specific regenerative medicine purposes mainly relies on their *in vitro* differentiation potency, which is often evaluated by qualitative approaches such as specific staining. More recently, molecular biology quantitative assays have been introduced although it clearly emerged that if the potency evaluation is biased by inaccurate data, suboptimal or poor results can be expected also for clinical purposes.

To obtain reliable and comparable results, the validation process and the comparison of molecular biology data remain controversial and open topics. For monitoring gene expression, quantitative real-time PCR (qRT-PCR) is often the method of choice, due to its sensitivity, large dynamic range, potential for high throughput, as well as accurate quantification, and high degree of potential automation. In the imperative need to obtain expression results that are not only accurate but also comparable among different experimental setups, conditions, operators and laboratories, normalization of qRT-PCR data should be performed against housekeeping genes (HGs), which must display unchanged expression in the cells irrespectively of the experimental conditions. It has to be noticed that very often the importance of selecting appropriate reference genes is not adequately emphasized [4] and the use of a single housekeeping gene without prior validation makes gene expression assays *via* qRT-PCR unreliable [5–8]. Thus, to overcome the bias introduced by suboptimal choice of reference genes, the new and optimal standard for gene expression analysis recommends the use of a single HG that has been validated for the process under study or, in the absence of this condition, that at least two HGs are used [9, 10].

Housekeeping genes considered suitable for qRT-PCR normalization are the ones present in all nucleated cell types, necessary for basic cell survival and considered stable in various tissues. These ‘traditional’ HGs include *GAPDH* (glyceraldehyde-3-phosphate dehydrogenase), *ALB* (albumin), *ACT*s (actins), *TUB*s (tubulins), *PPI*s (cyclophilins) and micro-globulins-encoding genes. Notably, several studies have already shown that for some cells the processes in which the above-mentioned genes are involved are not stable and their expression is therefore variable. For example, the cytoskeleton is modulated during culturing and the same happens for metabolic HGs, which are not only implicated in basal cell metabolism but also participate in other cell functions [11, 12]. To overcome such difficulties, the use of 18S or 28S ribosomal RNA (rRNAs) has become popular for validation and normalization, as the total amount of ribosomal RNA is generally assumed to be relatively stable. As rRNAs constitute about 80% of total RNA in the cells (and 18S+28S around 95% when RNAs are purified by anion exchange technology-based commercially available kits), equalizing the total RNAs input among different samples implies the assumption that the rRNA levels in the cells are unchanged. Unfortunately, recent studies have shown that the levels of ribosomal RNA do vary under experimental conditions [13, 14] thus making rRNAs unsuitable for normalization also because their very high levels make difficult to establish a reliable baseline value in most real-time PCR protocols.

In the recent years, very few studies in embryonic and human adult mesenchymal stem cells from different sources showed that the most popular reference genes appear to be unsuitable for normalization and validation as they may display varying expression levels [15–20]. The limited amount of data generated in these preliminary works is, to date, not yet sufficient to depict a clear and straight list of reliable HGs that could be used when studying MSCs under different growth conditions and differentiation processes. For these reasons, due to the availability in our laboratory of human MSCs isolated from adipose (AD)-, bone marrow (BM)- and cord blood (CB)-tissues, we compared the stability of a set of 12 traditionally used HGs after adipogenic, osteogenic and chondrogenic differentiation. Furthermore, we quantified the differential expression of adipogenic, osteogenic and chondrogenic markers to assess the effects of HG variability on their transcriptional patterns.

## Materials and methods

### Isolation and growth of MSCs

In this work, MSCs from bone marrow aspirate, lipoaspirate and human cord blood were obtained from healthy donors after informed consent.

#### Bone marrow mesenchymal stem cells (BMMSC)

Total bone marrow aspirate was directly seeded in alpha modified eagle medium (alpha MEM; Gibco-Life Technologies, Carlsbad, CA, USA) supplemented with 20% foetal bovine serum gamma irradiated (FBS; Gibco-Life Technologies) at the concentration of 50,000 WBC/cm^2^ in culture chamber. After 72 hrs, the supernatant was discarded and replaced by fresh complete medium. On day 14, colonies of MSCs were detached and re-seeded in the same culture conditions.

#### Adipose mesenchymal stem cells (ADMSC)

After centrifugation of lipoaspirate, the lower density solid phase was collected and treated with 0.075% collagenase (Roche Applied Science, Mannheim, Germany) in phosphate-buffered saline (PBS; Gibco-Life Technologies) for 30 min. at 37°C with gentle agitation. After collagenase inactivation, the stromal vascular fraction was re-suspended overnight in Dulbecco's Modified Eagle Medium-High Glucose (DMEM-HG, Gibco-Life Technologies) supplemented with 20% FBS (Gibco-Life Technologies) and adherent cells were cultured.

#### Cord blood mesenchymal stem cells (CBMSC)

Buffy coat obtained by centrifugation of cord blood was harvested and incubated for 20 min. with RosetteSep enrichment cocktail by lineage-negative depletion (StemCell Technologies, Vancouver, Canada). After density gradient, mononuclear cells were collected and plated in alpha MEM plus 20% FBS and 2 mM L-glutamine (all from Gibco-Life Technologies). All subsequent studies on cell growth and differentiation were carried out in duplicate.

### Adipogenic, osteogenic and chondrogenic differentiation

ADMSC, BMMSC and CBMSC (passage 5) were seeded in six-well plates at a concentration of 2 × 10^4^ cells/cm^2^ in 2 ml of media and cultured in control medium [alpha MEM supplemented with 20% FBS (both from Gibco-Life Technologies) until 70–80% confluence. To induce osteogenesis and chondrogenesis, the medium was switched to specific induction medium (Lonza GmbH, Cologne, Germany), supplemented with 10 ng/ml transforming growth factor β1 (TGFβ1) for chondrogenesis. Induction medium was changed every 3/4 days. After 3 weeks, cells were harvested separately from duplicate wells and another well was used for histochemical staining. To induce adipogenesis, the culture medium was switched to Lonza adipogenic induction medium for 4 days and then to Lonza adipogenic maintenance medium for 3 days, and this procedure was repeated three times (3 weeks) based on manufacturer's instructions. On the fourth week, the cells were cultured in only adipogenic maintenance medium with one intermediate change. Cells were then harvested from duplicate wells and another well was used for histochemical staining.

### Histochemical stainings

To detect adipogenesis, cells were fixed in 4% formaldehyde for 1 hr, washed repeatedly with PBS and then stained for 15 min. with fresh Oil-Red O solution (Sigma-Aldrich Inc, St Louis, MO, USA; three parts of a 0.5% stock solution in isopropanol and two parts of distilled water) and washed three times with distilled water. To assess calcium accumulation during osteogenesis, cultures were rinsed in PBS, fixed in ice-cold 70% ethanol and incubated with Alizarin red solution (2 g/100 ml in distilled water, Sigma-Aldrich) for 15 min., after which the wells were rinsed repeatedly with water. Chondrogenesis was confirmed using the stain Alcian blue (1 g/l in 0.1 M HCl, Sigma-Aldrich) for 30 min. at room temperature. Before staining, the chondrogenic cultures were fixed in 4% formaldehyde for 15 min. and washed with several changes of PBS.

### RNA isolation and cDNA synthesis

Total RNA was isolated using the RNeasy Mini kit (Qiagen, Hilden, Germany). On-column DNase digestion of the samples was performed following manufacturer's instructions. From each sample, two biological replicates were processed. The purity of the RNA was determined by measuring the absorbance A_260_/A_280_ in a Nanodrop spectrophotometer. RNA integrities were assessed using electrophoretic techniques. First strand cDNAs were synthesized from 500 ng of total RNA in 20 μl final volume, using the iScript cDNA synthesis kit (Bio-Rad Laboratories, Hercules, CA, USA) according to the manufacturer's instructions.

### Quantitative RT-PCR assays

The primers for the reference genes used in this study were designed in-house using the NCBI Primer Designing Tool (http://www.ncbi.nlm.nih.gov/tools/primer-blast/), selecting only the primers spanning an exon–exon junction and producing a PCR amplificate with length between 70 and 150 base pairs (Table 1). The same tool and settings were used to generate the primers for the analysis of the stem cells differentiation, *ADIPSIN*, *OPN* and *COL10A1*. Primer sequences will be provided on request.

**Table 1:** Candidate reference genes analysed in this study

Gene	UniGene	GenBank	Symbol	Description
1	N/A	X03205.1	18SrRNA	Human 18S ribosomal RNA
2	Hs.520640	NM_001101.3	*ACTB*	Actin, beta
3	Hs.534255	NM_004048.2	*B2M*	Beta-2-microglobulin
4	Hs.356331	NM_021130.3	*PPIA*	Peptidylprolyl isomerase A
5	Hs.544577	NM_002046.3	*GAPDH*	Glyceraldehyde-3-phosphate dehydrogenase
6	Hs.255230	NM_000181.3	*GUSB*	Glucuronidase, beta
7	Hs.586423	NM_001402.5	*EF1alpha*	Eukaryotic translation elongation factor 1 alpha 1
8	Hs.590872	NM_003194.4	*TBP*	TATA-binding protein
		NM_001172085.1		
9	Hs.546356	NM_012423.2	*RPL13A*	Ribosomal protein L13a
10	Hs.546285	NM_001002.3	*RPLP0*	Ribosomal protein, large, P0
		NM_053275.3		
11	Hs.492407	NM_003406.3	*YWHAZ*	Tyrosine 3-monooxygenase/tryptophan 5-monooxygenase activation protein, zeta polypeptide
		NM_145690.2		
		NM_001135699.1		
		NM_001135700.1		
		NM_001135701.1		
		NM_001135702.1		
12	Hs.520348	NM_021009.5	*UBC*	Ubiquitin C

Real-time quantitative PCR assays were carried out in a BioRad CFX96 Real-Time PCR Detection System instrument (Bio-Rad Laboratories) using standard PCR conditions. Triplicates of all reactions were analysed and within each triplicate values exceeding S.D. >10% were discarded. Each assay also included a blank. To confirm product specificity, a melting curve analysis was performed after each amplification. Quantifications were performed using the ‘SsoFast EvaGreen Supermix’ (Bio-Rad Laboratories).

For quantification of differentiation, the abundance of the transcripts in induced cells was determined relative to the standard reference genes selected for each differentiation process and with respect to the control cells in alpha MEM by using the Comparative Ct Method. For statistical analysis and expression data generation, the Bio-Rad CFX Manager software was used.

### geNorm Analysis

To evaluate the stability of candidate reference genes expressed as Ct values, we used geNorm v. 3.5. geNorm requires the transformation of Ct values by the 2^−ΔΔCt^ method, using the lowest Ct as a calibrator. geNorm computes all possible average pairwise variations between the candidate gene transformed Ct values and provides a measure of the expression stability (M) of each gene. An M-value below 1.5 identifies stable reference genes. geNorm then performs stepwise exclusion of the gene with the highest M-value (least stably expressed gene) and recalculates M-values for the remaining genes. This iterative process enables to rank candidate genes based on their stability of expression. As a single reference gene may not allow adequate normalization, geNorm computes the optimal number of reference genes required for accurate normalization by calculating V_n/n+1_ pairwise variations between consecutively ranked normalization factors NF_n_ and NF_n+1_, where n and n+1 are the number of genes considered, and NF_i_ are the geometric means of the i best candidate reference gene transformed Ct values. A pairwise variation of 0.15 is suggested as a cut-off value below which the inclusion of an additional reference gene is not required for reliable normalization [9].

### Normfinder analysis

To calculate the stability value with NormFinder program, for each gene the average Ct value of each duplicate reaction was converted to relative quantity data as described for geNorm [21]. The NormFinder reference tool was applied to rank the candidate reference gene expression stability for all samples with no subgroup determination. According to the analysis, the lowest stability value is top ranked.

## Results

### MSC differentiation properties and RNA quality control

**Fig. 1:**
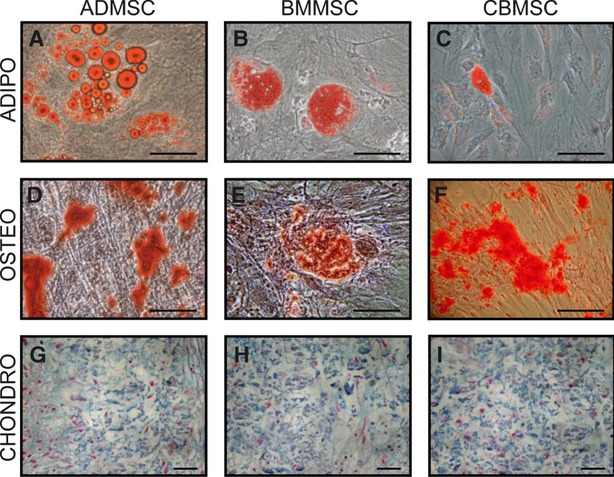
Histochemical staining of adipose-, bone marrow- and cord blood-derived MSCs subjected to adipogenic, osteogenic and chondrogenic differentiation. MSCs were cultivated in alpha MEM + 20% FBS and when cells reached 70–80% confluence, specific differentiation media were added (see Materials and methods). At the end of the induction, the degree of differentiation was evaluated by histochemical staining. (A–C) The intracellular accumulations of lipid droplets were stained with Oil-Red O (D–F) the osteogenic induction was unveiled by Alizarin red staining, which marks calcium-rich mineral deposits (G–I) the chondrogenic phenotype was proved by Alcian blue staining of the chondrocyte-specific glucosaminoglycans. Bar scale in each panel: 20 μm.

The MSCs were obtained from bone marrow aspirate, lipoaspirate and cord blood. Immunophenotype profile showed that they expressed the typical MSC cell-surface antigens, such as CD90, CD73, CD44 and CD105, and were negative for hematoendothelial markers such as CD34, CD133 and CD45 (data not shown). At passage 5, they were induced *in vitro* to differentiate towards osteocytes, chondrocytes and adipocytes. Cells under normal culture conditions maintained an undifferentiated phenotype with a marked fibroblast-like morphology, whereas under specific induction conditions both morphological changes and specific stainings demonstrated chondrogenic, osteogenic and adipogenic differentiation. In particular, for each cell type, Alizarin Red staining (osteogenesis) indicated calcium nodule formation and matrix mineralization, Alcian Blue staining (chondrogenesis) demonstrated synthesis of proteoglycans and Oil-Red O staining (adipogenesis) showed lipid droplets formation (Fig. 1). Regarding adipogenic potential, ADMSC gave rise to the highest degree of differentiation with respect to BMMSC and especially to CBMSC. These results confirmed that isolated MSCs exhibit mesenchymal features.

**Fig. 2:**
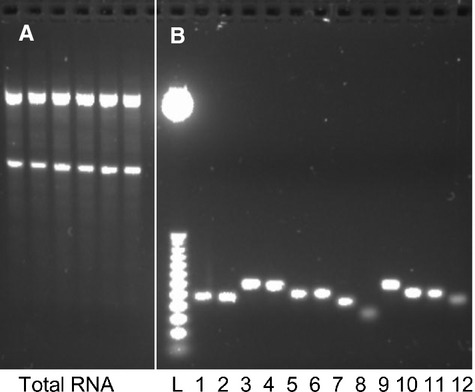
Total RNA quality and specificity of primers. (**A**) 100 ng of total RNA from six random samples were loaded per lane. All samples show complete absence of degradation and high degree of integrity. (**B**) Primers specific for the 12 genes used in the study have been checked for their amplification specificity on cDNA obtained from a CBMSC sample. All couples of oligonucleotides gave rise to a single band of the expected length. Lane L = 100 bp Ladder; from Lane 1 to 12: *PPIA*, *GUSB*, *TBP*, *RPLP0*, *YWHAZ*, *EF1alpha*, *RPL13A*, *UBC*, *B2M*, *ACTB*, *GAPDH* and 18S rRNA.

For each condition, the cells were cultured and harvested in duplicate at the beginning and at the end of the inductions. The cells were also let to grow under normal condition in a separate culture and harvested at the same time of the end of the three differentiation processes. RNA was extracted from 30 samples and the yield of isolated RNA varied from 1.25 to 34 μg. By spectrophotometric analysis, we assessed that the purity of the samples was suitable for further analysis, with a mean A_260_/A_280_ ratio of 2.06. Finally, the quality and integrity of extracted RNA was assayed in six random samples by agarose gel electrophoresis that showed absence of ribosomal RNA degradation with a 28S/18S rRNA amount ratio around 2 (Fig. 2A).

### Expression levels and homogeneity of candidate HGs

For the selection of the HGs, we analysed for each sample the genes coding for beta-actin (*ACTB*), beta-2-microtubulin (*B2M*), eukaryotic translation elongation factor 1 alpha 1 (*EF1alpha*), glyceraldehyde-3-phosphate dehydrogenase (*GAPDH*), beta-glucuronidase (*GUSB*), cyclophilin A (*PPIA*), 60S basic ribosomal protein L13a (*RPL13A*), 60S acidic ribosomal protein P0 (*RPLP0*), TATAA-box-binding protein (*TBP*), ubiquitin C (*UBC*), tyrosine 3/tryptophan 5-monooxygenase activation protein (*YWHAZ*), and 18S rRNA (*RNR1*). Each couple of specific primers allowed the identification of only one specific DNA amplificate of the expected length (Fig. 2B).

**Fig. 3:**
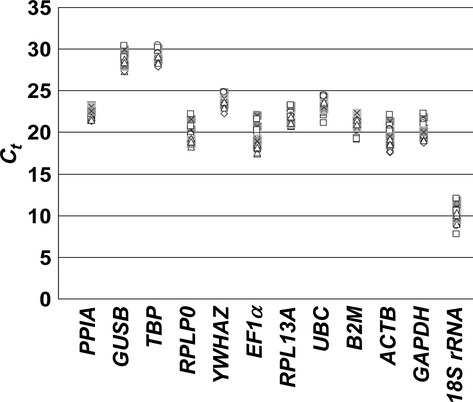
Raw quantitative qRT-PCR cycle threshold (Ct) for the 12 candidate housekeeping genes in the 30 MSCs samples. The range of the expression level of the 12 genes in the 30 samples is expressed as Ct values. Legend: rumble = time 0 samples; circle = osteogenic samples; open square = chondrogenic samples; closed square = adipogenic samples; triangle = time 3-week samples.

The 12 HG exhibited a broad array of expression level, from the lowest mean Ct of 10.1 for the 18S rRNA gene (*RNR1*) to the highest mean Ct of 29.1 for *TBP* (Fig. 3). Of the 12 genes, 11 showed a standard deviation (SD) ≤5% with the only exception of 18S rRNA gene, whose SD was 9%. This may be explained by the high 18S rRNA levels that make difficult to obtain a reliable baseline value. Therefore, the accuracy of the final data after analysis might be poor for this gene.

For each gene, to compare the homogeneity of the qRT-PCR values obtained from the 30 analysed samples, we performed Grubbs' test (significance level set as *P* ≤ 0.01 – two sided), also called the ESD method (extreme studentized deviate). This analysis allows determining whether the most extreme values in a given group are significant outliers, thus pointing out the values that are unlikely to have come from the same Gaussian population as the other values in the cluster. Notably, no outliers were detected and thus no samples were excluded from further analysis.

### Determination of HGs expression stability

Reference gene stability was evaluated using the geNorm and NormFinder VBA applets. The geNorm program calculates the expression stability (M) of a gene based on the average pairwise variation (V) between all studied genes, whereas NormFinder assigns a stability value to each candidate reference gene using a model-based approach [9, 21]. On one hand, the geNorm algorithm is highly dependent on the assumption that the genes being analysed are not co-regulated, and thus, as a pairwise comparison approach is used, co-regulated genes belonging to the same pathway or system with a similar expression profile would obtain too good score. On the other hand, if the expression profiles suggest that several candidate genes are co-regulated, a model-based evaluation method such as NormFinder should be considered. In absence of literature data about the possible co-regulation of the studied genes, we have chosen to use both applets for the analysis and to compare the outcome.

**Fig. 4:**
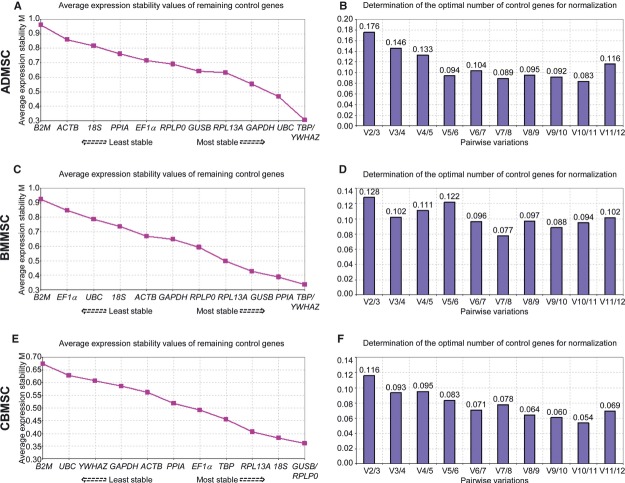
Evaluation of most stable reference genes and minimal number of genes for normalization across all the induction conditions for each cell line separately. (**A**, **C**, **E**) The genes were serially excluded from the analysis, with M representing the mean pairwise variation between an individual gene and all other tested control genes. The gene indicated at each point on the *x*-axis is the one that is to be excluded from the following step. The most stable genes are those that are still included, that is, those that exhibit the lowest M. (**B**, **D**, **F**) The pairwise variation V of the normalization factors was calculated for the different cell lines for the 12 housekeeping genes with the geNorm software. Pairwise variation value below 0.15 with the least number of reference candidates used is considered optimal.

**Fig. 5:**
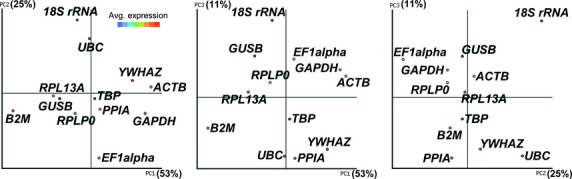
Population-specific principal component analysis (PCA) of gene expression in cord blood mesenchymal stem cells during differentiations, to check if analysed genes group together. Gene names are presented in the figure. PCA applied to the entire data set (components 1, 2 and 3) model explaining *r*^2^ = 89% of the data variation. Centred data.

The qRT-PCR data generated in our experimental standard and differentiation conditions allowed us to analyse HGs stability from different points of view. First, we decided to identify the most stable reference genes across all the tested conditions (adipogenic, chondrogenic and osteogenic differentiations) for every cell line separately. Remarkably, using the geNorm method, all 12 genes under examination reached high expression stability, with low M (expression stability index, see Methods for details), that is, below the default limit of 1.5 (Fig. 4). The analysis showed that for ADMSC and BMMSC, the genes that varied the least their expression levels were *TBP* and *YWHAZ*, whereas for CBMSC *GUSB* and *RPLP0* resulted the most stable ones. Interestingly, *B2M* expression varied the most in all MSCs. Moreover, the optimal number of reference genes was determined calculating the pairwise variation (V) between a given number of HG and the inclusion of an additional gene. A cut-off value of 0.15 has been suggested, where the inclusion of an extra gene has little effect on the normalization (see Methods for a more detailed explanation). For BMMSC and CBMSC, the inclusion of three genes as opposed to two (V2/3 are 0.128 and 0.116, respectively) would have a small effect for normalization, whereas for ADMSC, the minimal number of HG to be used should be three as V3/4 is 0.146 and V2/3 is 0.176 (and thus *UBC* should be added). Performing the same stability analysis with the alternative VBA applet NormFinder, *RPL13A* was the most stable gene in ADMSC, followed by *GUSB* and *GAPDH* (Table 2). *TBP*/*YWHAZ*, suggested as the two top-ranking genes with geNorm, were ranked number 4 and 6. Both geNorm and NormFinder ranked the commonly used 18S rRNA, *ACTB* and *B2M* as the least suitable reference genes in ADMSC. In BMMSC, *GUSB* and the two best geNorm genes *YWHAZ* and *TBP* were ranked as the most suitable normalization genes, whereas 18S rRNA, *EF1alpha* and *B2M* again had the least stable expression profiles together with *UBC* (Table 2). *TBP* ranked best in CBMSC followed by *RPLP0* (the best geNorm gene with *GUSB*) and, again, *B2M* was the least stable gene as for geNorm analysis (Table 2). From these data, it emerges that there are small discrepancies between the two algorithms, with *TBP*, *YWHAZ* and *GUSB* being among the best ranking genes analysed with both methods. To verify that the reference genes are not co-regulated (basic assumption for the validity of the geNorm analysis), gene distribution patterns were investigated with principal component analysis (PCA; lgsun.grc.nia.nih.gov/ANOVA/index.html) [22]. Notably, for all the sources, the genes did not group tight together through the PCA plots (Fig. 5 for representative plots of the first three principal components for CBMSC) and no PC-based gene clusters were found according to their contribution to the principal components (fold-change threshold for clusters = 1.5; correlation threshold for clusters = 0.7).

**Table 2:** NormFinder ranking of candidate genes by source

	ADMSC–ALL. DIFF.			BMMSC–ALL. DIFF.			CBMSC–ALL. DIFF.		
			
Rank	Gene	StVal	GeNorm	Gene	StVal	GeNorm	Gene	StVal	GeNorm
1	*RPL13A*	0.199	5	*GUSB*	0.144	4	*TBP*	0.119	5
2	*GUSB*	0.262	6	*YWHAZ*	0.172	1	*RPLP0*	0.169	1
3	*GAPDH*	0.283	4	*TBP*	0.256	1	*18S*	0.236	3
4	*TBP*	0.338	1	*PPIA*	0.280	3	*PPIA*	0.251	7
5	*UBC*	0.348	3	*RPL13A*	0.352	5	*RPL13A*	0.320	4
6	*YWHAZ*	0.356	1	*GAPDH*	0.420	7	*GUSB*	0.323	1
7	*RPLP0*	0.475	7	*ACTB*	0.432	8	*EF1alpha*	0.329	6
8	*EF1alpha*	0.535	8	*RPLP0*	0.481	6	*ACTB*	0.372	8
9	*PPIA*	0.545	9	*18S*	0.571	9	*UBC*	0.396	11
10	*18S*	0.615	10	*UBC*	0.575	10	*GAPDH*	0.407	9
11	*ACTB*	0.678	11	*EF1alpha*	0.755	11	*YWHAZ*	0.429	10
12	*B2M*	0.955	12	*B2M*	0.835	12	*B2M*	0.569	12

**Fig. 6:**
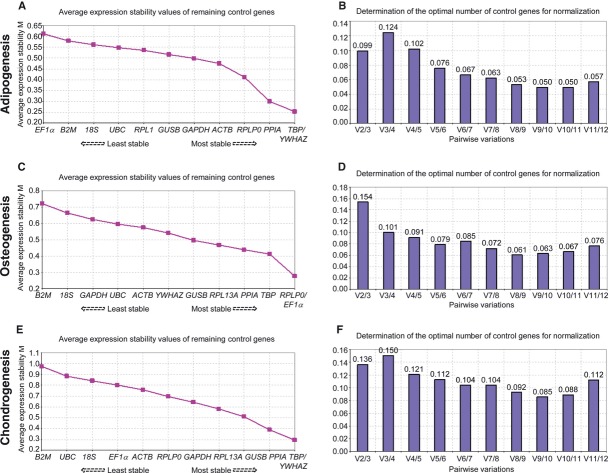
Evaluation of most the stable reference genes and the minimal number of genes for normalization across all cell lines for each differentiation process separately. (**A**, **C**, **E**) Average expression stability values (M) of 12 most stable reference candidates after stepwise exclusion of the least stable reference candidate. M-values are represented. (**B**, **D**, **F**) Pairwise variation analysis showing optimal number of reference candidates for normalization.

After this analysis, we decided to examine the qRT-PCR data from the point of view of the adipogenic, osteogenic and chondrogenic processes. Therefore, we checked the stability of the selected HGs with respect to the single differentiation, but regardless of the cell source. geNorm identified, as most stable reference genes, *TBP*/*YWHAZ* for adipogenesis and chondrogenesis, and *RPLP0*/*EF1alpha* for osteogenic induction (Fig. 6). Moreover, the V value showed that the inclusion of three genes instead of two would have a small effect for adipogenic and chondrogenic process evaluation (V2/3 of 0.099 and 0.136, respectively), whereas it would improve the analysis for osteogenesis (V3/4 of 0.101 *versus* V2/3 of 0.154), with the inclusion of *TBP* (Fig. 6). NormFinder analysis pointed out that: (a) for adipogenesis *RPLP0* was the most stable gene, followed by *GUSB* and *TBP*, whereas 18S rRNA, *B2M* and *EF1alpha* ranked as the least stable genes (Table 3); (b) for the osteogenic process, the geNorm best housekeeping *TBP* and *RPLP0* together with *PPIA* were ranked as the most suitable normalization genes, whereas the commonly used *GAPDH*, 18S rRNA and *B2M* had the least stable expression profiles (Table 3); (c) finally, for chondrogenesis, *GUSB* together with the two best geNorm genes *TBP* and *YWHAZ* resulted the most stable ones with *B2M* again as least stable (Table 3). Summarizing the data generated by geNorm and NormFinder for the single differentiation processes analysed separately, the most stable reference genes resulted to be *TBP/YWHAZ* (geNorm) and *GUSB*/*RPLP0* (NormFinder), whereas *B2M* exhibited the least stable expression profiles. PCA showed absence of co-regulation (data not shown).

**Table 3:** NormFinder ranking of candidate genes by differentiation processes

	ALL–ADIPOGENESIS			ALL–OSTEOGENESIS			ALL–CHONDROGENESIS		
			
Rank	Gene	StVal	GeNorm	Gene	StVal	GeNorm	Gene	StVal	GeNorm
1	*RPLP0*	0.195	4	*RPLP0*	0.213	1	*GUSB*	0.186	4
2	*GUSB*	0.204	7	*PPIA*	0.230	4	*TBP*	0.195	1
3	*TBP*	0.206	1	*EF1alpha*	0.245	1	*YWHAZ*	0.269	1
4	*RPL13A*	0.289	8	*TBP*	0.276	3	*RPL13A*	0.352	5
5	*YWHAZ*	0.292	1	*GUSB*	0.292	6	*GAPDH*	0.359	6
6	*PPIA*	0.306	3	*RPL13A*	0.321	5	*PPIA*	0.387	3
7	*ACTB*	0.307	5	*UBC*	0.333	9	*18S*	0.501	10
8	*UBC*	0.308	9	*ACTB*	0.349	8	*RPLP0*	0.518	7
9	*GAPDH*	0.308	6	*YWHAZ*	0.374	7	*UBC*	0.577	11
10	*18S*	0.324	10	*GAPDH*	0.452	10	*ACTB*	0.601	8
11	*B2M*	0.395	11	*18S*	0.497	11	*EF1alpha*	0.708	9
12	*EF1alpha*	0.469	12	*B2M*	0.626	12	*B2M*	0.924	12

**Fig. 7:**
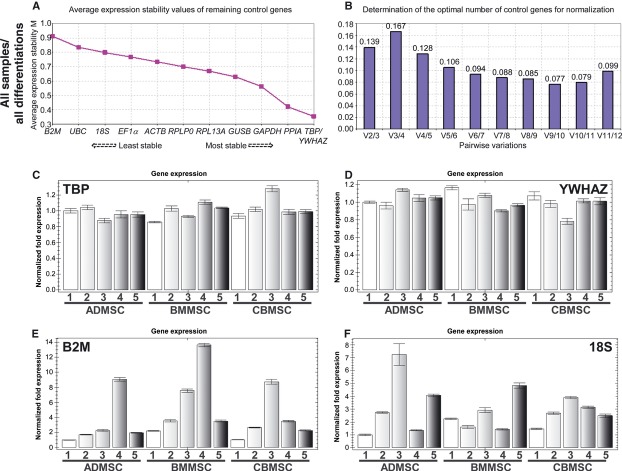
Average expression stability and optimal number of control genes for normalization across all the samples. (**A**) geNorm ranking of stability of genes for all the samples under study, with *B2M* being the least stable gene (highest M-value) and *TBP* and *YWHAZ* the most stable genes. (**B**) Pairwise variability plot. The use of the two most stable genes, V2, is sufficient for an accurate normalization (cut-off of 0.15). Each bar represents change in normalization accuracy when stepwise adding more endogenous controls according to ranking in panel A. (**C**–**F**) Expression levels of the two most stable reference genes identified in a and of two of the most popular housekeeping genes, *B2M* and 18S rRNA, normalized to the geometric mean of *TBP* and *YWHAZ* (Legend: 1 = time 0 samples; 2 = adipogenic samples; 3 = osteogenic samples; 4 = chondrogenic samples; 5 = time 3-week samples).

Finally, we ranked candidate HGs stability taking into account all the 30 samples under evaluation. This approach permits a comparative analysis across HGs and allows defining the most stable reference genes regardless of the source or the differentiation. geNorm method ranked *TBP* and *YWHAZ* as the two most stable genes (Fig. 7A). Concerning the widely used ‘gold standard’ 18S rRNA, *B2M* and *GAPDH*, the first two appeared the less stable genes, whereas the third resulted to fall among the most stable ones (Fig. 7A). The V factor calculated by geNorm indicated that adding a third gene to the most stable two would not be necessary (V2/3 = 0.139), although including nine HGs showed the smallest variation (V9/10 of 0.077) (Fig. 7B). Moreover, to show the consistent divergences in stability between the reference genes in our study when all samples are analysed together, we normalized the most commonly used reference genes 18S rRNA and *B2M* as well as the two most stable genes *TBP* and *YWHAZ* to the geometric mean of *TBP* and *YWHAZ* themselves. As shown in Figure 7C–F, the levels of both *B2M* and 18S rRNA randomly increase during the differentiation processes, without a common pattern linked either to the differentiation or to the tissue origin, depicting clearly their inconsistency as reliable HGs. These data were confirmed by the NormFinder analysis that ranked *TBP* and *YWHAZ* together with *GUSB* as the most stable reference genes, with *B2M* showing the least stable expression profile (Table 4). Finally, PCA plotting exhibited a high dispersion of the genes under analysis, thus confirming their high degree of independency (data not shown).

**Table 4:** NormFinder ranking of candidate genes regardless of cell sources and differentiations

	ALL–ALL. DIFF.		
	
Rank	Gene	StVal	GeNorm
1	*GUSB*	0.235	5
2	*TBP*	0.248	1
3	*YWHAZ*	0.316	1
4	*RPL13A*	0.355	6
5	*PPIA*	0.366	3
6	*GAPDH*	0.374	4
7	*RPLP0*	0.453	7
8	*18S*	0.488	10
9	*ACTB*	0.495	8
10	*UBC*	0.531	11
11	*EF1alpha*	0.617	9
12	*B2M*	0.815	12

### Dramatic effect of choosing suboptimal HGs

**Fig. 8:**
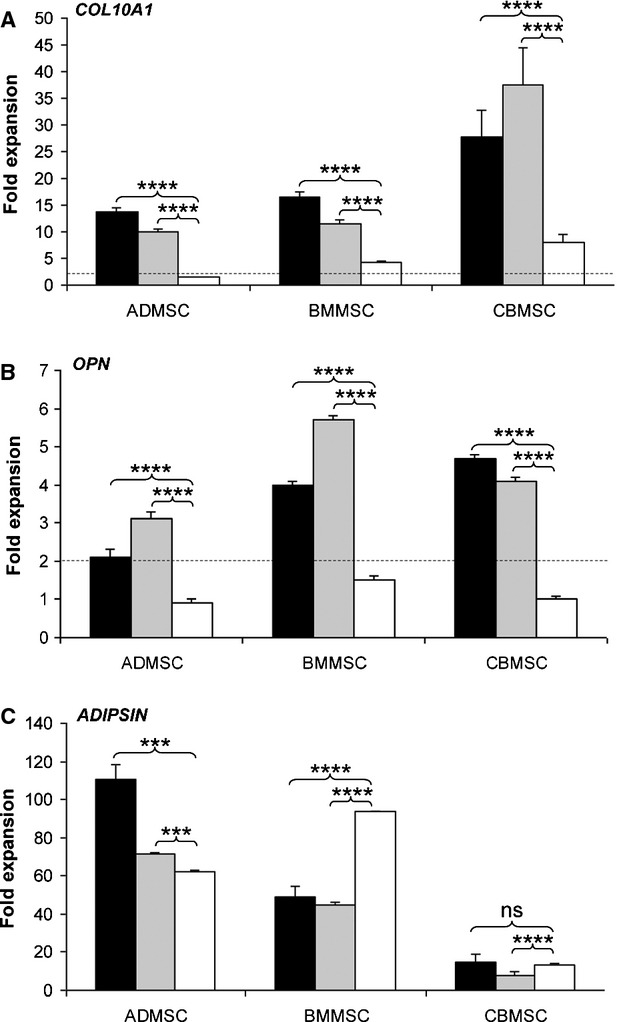
The effect of suboptimal choice of the housekeeping genes on the relative gene expression levels of the chondrogenesis marker *COL10A1*, osteogenesis marker *OPN* and the adipogenesis marker A*DIPSIN*. (**A**) The expression level of *COL10A1* is presented after chondrogenic induction relative to values at beginning of differentiation. The values have been normalized to either the geometric mean of *TBP* and *YWHAZ* (black bars), or *GUSB* (light grey bars) or *B2M* (white bars). (**B**) The result of osteogenic differentiation on the expression of *OPN* is shown after values have been normalized to either the geometric mean of *RPLP0* and *EF1alpha* (black bars), or *RPLP0* (light grey bars) or *B2M* (white bars). (**C**) The effect of adipogenic induction on the expression of *ADIPSIN* is presented after the values have been normalized to either the geometric mean of *TBP* and *YWHAZ* (black bars), or *RPLP0* (light grey bars) or *EF1alpha* (white bars). Error bars express the standard deviation of the mean. *P*-values have been obtained performing unpaired *t*-test. ****P ≤ 0.0001, ***P ≤ 0.001 and ‘ns’ stands for a *P* ≥ 0.05.

In many gene expression stability evaluations, the choice of suboptimal reference genes can generate misleading results. This is especially true for the analysis of genes whose expression slightly varies between control and treated samples. To illustrate the effect generated by normalizing to stable or unstable HGs, we analysed (a) the expression levels of the chondrocyte-specific alpha chain of type X collagen coding gene *COL10A1* after chondrogenic induction [23]; (b) the transcript levels of the *OPN* gene, coding for a highly phosphorylated sialoprotein (Osteopontin) that is a prominent component of the mineralized extracellular matrices of bones, after osteogenic differentiation [24]; (c) the variation in the expression of the adipogenic-specific gene *ADIPSIN* after adipogenic stimulation [25]. The transcript levels were normalized to the geometric mean of the two most stable genes suggested by geNorm method or to the top-ranking gene generated by NormFinder for each differentiation process regardless of the source of the cells, as well as to the least stable *B2M* (chondrogenesis and osteogenesis) or *EF1alpha* (adipogenesis). As shown in Fig. 8A and B, for both chondrogenesis and osteogenesis the use of the most unstable *B2M* gene gave rise to a dramatic effect on the correct evaluation of gene expression for all the MSCs under examination. In many cases, the bias introduced by the use of this suboptimal reference gene resulted in the apparent absence or strong reduction of differentiation potential, thus rendering real-time analysis completely unreliable. Regarding adipogenic differentiation, the use of *EF1alpha* did not lead to a complete loss of information as *ADIPSIN* resulted to increase its expression level with respect to the control for all sources (Fig. 8C). Indeed, *EF1alpha* instability did not allow a reliable comparison of the lineage potential properties among the three mesenchymal cell lines. In fact, when *EF1alpha* was used as HG, BMMSC showed an apparent higher *ADIPSIN* levels than adipose-derived cells, this being in contrast with the identical results obtained both with the analysis performed with the most reliable HGs (Fig. 8C) and with the histochemical staining (Fig. 1), demonstrating a more pronounced ADMSC adipogenic potential with respect to BMMSC and CBMSC.

## Discussion

The development of therapeutic approaches using MSCs is currently constrained by the lack of knowledge about the *ex vivo* properties and potency of the native MSCs. In fact, although one of the criteria used to define MSCs is the ability to differentiate into the three mesenchymal lineages [26], that is, bone, cartilage and fat, not all the MSC types are able to efficiently differentiate with the same degree of lineage potential. Moreover, there is increasing evidence that MSC populations are heterogeneous with coexisting subsets having varying potency [27, 28]. In this regard, Karystinou and colleagues [29] recently reported that human synovium-derived clonal MSCs are variably capable to perform osteogenesis and chondrogenesis, whereas only 30% of them are able to differentiate into adipocytes. For these considerations, the validation of reliable and intra- and inter-laboratories comparable experimental approaches is needed to predict potency of human MSC preparations especially for clinical application. Gene expression quantification is considered an emerging method to confirm or confute effective differentiation potency, and, in this context, the choice of not strategically selected or highly validated reference genes may lead to inconsistent results [30].

In this work, we aimed to identify refined sets of HGs for differentiation studies with MSCs from different sources comparing, at our knowledge for the first time, the most studied and promising human adult mesenchymal stem cell lines for regenerative medicine. To obtain comparable results not influenced by different experimental settings, which is the main limit of the few publications available, we rigorously conducted the experiments in parallel by using the same supplier for both growing and differentiation media and for the plastics, using MSCs differentiating at the same passage and for exactly the same time period. With this experimental approach, we obtained highly homogeneous data, and thus, the genes we propose, which are expressed at relatively constant levels across different experimental conditions or cell lines, can be considered a reliable selection to be tested as internal controls for normalization of gene expression data in similar analysis. Moreover, we would like to stress that traditionally used HGs (*B2M* and 18S rRNA) in somatic cells and MSCs and often used as internal standards in commercial gene expression arrays were not found to perform well in all investigated sets of differentiating MSCs.

By analysing separately the three MSC sources under differentiation, we identified *TBP*/*YWHAZ* (geNorm) and *RPL13A* (NormFinder) for ADMSC, *TBP*/*YWHAZ* (geNorm) and *GUSB* (NormFinder) for BMMSC, and *GUSB*/*RPLP0* (geNorm) and *TBP* (NormFinder) for CBMSC as the best performing genes. Our geNorm results regarding ADMSC are in agreement with those proposed by Fink and coworkers [17] that analysed with the same VBA applet the same cell type under the three classical differentiation processes, then confirming our postulate that validated HGs can be used to generate comparable inter-laboratories data. Moreover, both in Fink *et al*. [17] and in a previous work [31] on preadipocytes and mature adipocytes exposed to different hormones, the mostly used and frequently available *GAPDH* was proposed to be relatively stable. In our study, we also observed this gene in a good position (fourth of 12) in the geNorm stability ranking and in third position for the NormFinder method. Regarding BMMSC, the only published results on HGs stability during differentiation were part of a study by the Lopez group [18] on osteogenic induction performed using completely different data analysis tools with respect to us. The authors proposed *RPL13A* as the most stable normalizer gene with respect to *GAPDH* and *ACTB*. Our data confirmed and extended the validity of their results. In fact, the inclusion of chondrogenic and adipogenic differentiations, together with a wider array of studied HGs, showed for both VBA applets a higher stability for *TBP* and *YWHAZ* with respect to *RPL13A*, which in our conditions lies in the average position of the stability ranking and again precedes *GAPDH* and *ACTB*. Finally, to our knowledge, our report is the first attempt to identify stable HGs in CBMSCs during differentiation. These cells showed a partially different gene signature than ADMSC and BMMSC with *YWHAZ* in the bottom positions of the ranking and *TBP/RPLP0* among the top positions for both geNorm and NormFinder.

If we consider all the samples under analysis regardless of the source or the differentiation process and in view of identifying stable HGs for human mesenchymal stem cells under differentiation, *TBP* and *YWHAZ*, with *GUSB,* resulted again to be among the best normalizers for both VBA applet methods, rendering in our opinion their use preferable if no studies have previously been performed on newly isolated similar cells. In this view, it is interesting to compare our data with those of the only other reference gene stability report that has been published for differentiating umbilical cord-derived MSCs [20]. Notably, although distinct experimental setups and growth/differentiating conditions were used between us and the Wang group, in both studies *YWHAZ* and *GAPDH* ranked in the best positions. Moreover, *GAPDH* has been already proposed to perform well in most systems related to mouse or human embryonic stem cells [16], thus rendering its use affordable and convenient, with respect to other popular ‘gold standard’ reference genes in studies in which no HGs validation can be performed.

Taking advantage of the identification of the best reference genes for each differentiation process regardless of the source of MSCs, we also measured the expression levels of osteocyte-, chondrocyte- and adipocyte-lineage genes (*OPN*, *COL10A1* and *ADIPSIN*) before and after differentiation. The data clearly demonstrate that a suboptimal choice of reference genes can lead, in the worst case, to a complete loss of information about differentiation potency of MSCs or, in a less dramatic case, to an erroneous comparison of lineage potentials. This approach of validating reference genes should be applied to all the preclinical protocols aimed to study potency of MSCs, where it can be expected that in addition to the properties that are intrinsic to the cell preparation, other factors such as inflammation or biomechanics will influence gene expression.

In conclusion, the use of the best-validated HGs is mandatory to compare results produced in different laboratories. The decision on the use of a specific cell type in a defined clinical context should be based on reliable, standardized and reproducible results. For this reason, the results herein presented are, in our opinion, of crucial importance not only for basic researchers who want to improve the knowledge on the biological properties of novel cell sources but also for those involved in realizing well-characterized cell therapy products with gold standard quality controls and potency assays.
